# Atherogenic Lipid Profile and Systolic Blood Pressure are Associated with Carotid Artery Intima-media Thickness in Children with Turner Syndrome

**DOI:** 10.4008/jcrpe.v1i2.9

**Published:** 2010-12-08

**Authors:** Özgür Pirgon, Mehmet Emre Atabek, Bülent Oran, Rıdvan Güçlü

**Affiliations:** 1 Division of Pediatric Endocrinology, Faculty of Medicine, Selcuk University, Konya, Turkey; +90-332-223 63 50+90-332-223 61 81pirgon@mynet.comSelçuk University Faculty of Medicine, Division of Pediatric Endocrinology Konya, Turkey

**Keywords:** children, Turner syndrome, cardiovascular disease, carotid intima-media thickness

## Abstract

**Objective**: Women with Turner syndrome (TS) have greater carotid artery intima-media thickness (cIMT) known to be a risk factor for atherosclerosis in adults. To determine whether there are risk factors for atherosclerosis in children with TS, we compared cIMT, anthropometric and metabolic parameters between children with TS and healthy controls.

**Methods**: Data of children with TS with XO karyotype (n=24, mean age: 11.6±3.6) were compared with those of healthy children (n=24, mean age: 10.5±3.6) with respect to anthropometric parameters, lipid levels, insulin resistance and cIMT which was measured by high resolution B-mode ultrasonography.

**Results**: Mean age and cIMT values were similar in the two groups of children. However in children with TS, fasting glucose (p=0.01), total cholesterol (p=0.006), triglyceride (p=0.04) levels and HDL-cholesterol (p=0.002) levels were higher than those of controls. In the TS group, cIMT correlated positively with LDL-cholesterol (r=0.435, p=0.034) and with systolic blood pressure (r=0.430, p=0.036) and negatively with HDL-cholesterol (r=-0.518, p=0.01). In stepwise regression analysis, HDL-cholesterol emerged as a significant predictor of cIMT (b= -0.518, p=0.01) contributing to 26.8 % of its variability.

**Conclusion**: The systolic blood pressure and dyslipidaemia were shown to be risk factors for atherosclerosis in children with TS.

**Conflict of interest:**None declared.

## INTRODUCTION

Turner syndrome (TS) is associated with insulin resistance, increased incidence of type 2 diabetes and hypertension, which all constitute cardiovascular risk factors. A survey of morbidity and mortality in patients with TS identified by a chromosomal defect registry found that cardiovascular diseases, congenital or acquired, were the single highest cause of death.(1, 2, 3)

Increased common carotid artery intimamedia thickness (cIMT) is significantly related to known cardiovascular risk factors and to carotid plaque, a more advanced atherosclerotic lesion.(4) Measuring cIMT with ultrasonography correlates well with pathological measurements and is reproducible. Increased cIMT is correlated with cardiovascular risk factors and the severity of coronary atherosclerosis and predicts cardiovascular events in population groups.(5, 6) Carotid artery IMT as a marker of early atherosclerosis has been studied using vascular ultrasonography in children with familial hypercholesterolemia(7), diabetes(8, 9), hypertension(10) and childhood obesity.(11, 12) Although the clinical complications of atherosclerosis such as coronary artery disease and stroke usually occur in middle and late ages, autopsy studies have shown that the atherosclerotic process in the vascular wall begins in childhood and is accelerated in the presence of risk factors.(13, 14) Clustering of cardiovascular risk factors is seen in children and adolescents with the highest degree of insulin resistance, suggesting that adult cardiovascular disease is more likely to develop in these young people.(15, 16)

Increased mortality and morbidity have been reported in TS, and possibly this is in relation to an increased frequency of diabetes and cardiovascular diseases.(3, 17) Type 2 diabetes mellitus, insulin resistance, and impaired glucose tolerance are also relatively more frequent findings in TS patients. However, no study has been conducted to investigate the relationship between cardiovascular risk factors such as insulin resistance, glucose intolerance, dyslipidaemia, hypertension and cIMT in children with TS. In the present study, we investigated the relationships between the risk factors of atherosclerosis and cIMT and insulin sensitivity indices based on fasting samples in children with TS.

## MATERIALS AND METHODS

**Subjects**

The TS group consisted of twenty-four girls (mean age: 11.6±3.6 years) with newly diagnosed TS on the basis of karyotype analysis. These children were recruited from short children who attended the outpatient clinic of the Pediatric Endocrinology Unit of Selcuk University Hospital in Konya, Turkey. The clinical diagnosis of TS was based on peripheral leucocyte karyotype analysis and all subjects included in the study had 45, XO karyotypes. The other karyotype forms were excluded. Subjects with thyroid dysfunction and celiac disease were also excluded from the study. None of the patients were on any medication such as growth hormone or estrogen therapy.

Control subjects (24 girls; mean age: 10.5±3.6 years) were healthy children who presented to the hospital for minor illnesses such as common cold. We collected blood samples of the control group at the time of the follow-up examination after resolution of their minor illnesses. Children were excluded if they had prior major illness including type 1 or type 2 diabetes, were on medications, or had a condition known to influence body composition, insulin action, or insulin secretion (e.g. glucocorticoid therapy, hypothyroidism, Cushing’s disease, growth hormone or estrogen therapy). The study was approved by the local ethics committee of the Selcuk University. Signed informed consent was obtained for each subject over 12 years of age, and informed parental consent was also obtained for all children regardless of age.

Each child underwent a complete physical examination, including anthropometric measurements. Height was measured to the nearest 0.5 cm on a standard height board, and weight was determined to the nearest 0.1 kg on a standard physician’s beam scale with the subject dressed only in light underwear and no shoes. Waist circumference was measured at the level of the umbilicus with the patient standing and breathing normally. Hip circumference was measured at the level of the iliac crest. Body mass index (BMI) was calculated as weight (in kilograms) divided by height (in meters) squared [weight (kg)/height (m)^2^]. Blood pressure was measured with a standard mercury sphygmomanometer after the subject had rested for at least 10 minutes.

**Biochemical analyses**

Blood samples were taken in the morning between 7:30 and 9:30 A.M. after the children had fasted overnight. After clotting, the serum was separated and biochemical investigations, except for insulin, were performed immediately. A serum sample was deep-frozen for insulin measurement. Glucose was determined by the glucose oxidase method. Plasma concentrations of total cholesterol, high-density lipoproteincholesterol (HDL-cholesterol), low-density lipoprotein-cholesterol (LDL-cholesterol) and triglycerides were measured using a routine enzymatic method with Olympus 2700 Analyzer (Olympus Diagnostica GmbH, Ireland). Insulin levels were measured by an IMMULITE immunoassay (IMMULITE Diagnostic Products Corporation, Los Angeles, CA). Sensitivity and specificity of the insulin kit were 92.86% and 87.01%, respectively.

**Insulin indices derived from fasting blood samples**

The homeostasis model assessment of insulin resistance (HOMA-IR), quantitative insulin- sensitivity check index (QUICK-I) and fasting glucose-to-insulin ratio (FGIR) were derived as estimates of insulin resistance. FGIR was calculated as fasting insulin concentration (μU/mL)/fasting glucose concentration (mg/dL). HOMA-IR was calculated as fasting insulin concentration (μU/mL) X fasting glucose concentration (mmol/L)/22.5.(18) QUICK- I was calculated as 1/[(log fasting insulin concentration (μU/mL) + log fasting glucose concentration (mg/dL)].(19)

**Ultrasound imaging**

All patients were examined by a pediatric cardiologist who was blinded to the participants’ laboratory values and risk factor levels. After routine cardiovascular examination, a chest roentgenogram and electrocardiogram were obtained in all patients. Scans were obtained at rest; the subjects were laid quietly for 10 minutes before the first scan. A Philips Sonos 5500 system with a 7-12 MHz transducer ultrasonic imager was used for echocardiographic assessments. The measurements were obtained using the published standards recommended by the American Society of Echocardiography.(20) The participants were examined in the supine position with the head turned slightly to the left. Longitudinal images of the common carotid artery were obtained by combined B-mode and color Doppler ultrasound examinations. The cIMT of the common carotid artery posterior (far) wall was measured with the electronic calipers of the machines, as described by Pignoli et al.(21) On a longitudinal, twodimensional ultrasound image of the carotid artery, the far wall of the carotid artery was displayed as two bright white lines separated by a hypoechogenic space. The distance between the leading edge of the first bright line of the far wall and the leading edge of the second bright line indicated the cIMT. The cIMT was measured during end diastole. The cIMT measurements were performed on-line. The mean cIMT was calculated for each child as the average of three consecutive measurements of maximum far wall thickness obtained from the common carotid artery, 20 mm below the carotid bulb. The coefficients of variation of the measurements were less than 3%.

**Statistical methods**

The data were expressed as means±SD. The Kolmogorov-Smirnov test was applied separately for groups to check the normality of the variables. Differences in the means of variables were tested using both parametric and non-parametric tests depending on the distribution of the variables. Triglyceride, fasting insulin, FGIR and HOMA-IR variables (not normally distributed) were log-transformed before data analysis. Statistical correlation was assessed using the Pearson test (r). Separate relationships between cIMT and insulin sensitivity indices (HOMA-IR, FGIR and QUICK-I) were also examined after adjustment for age, sex, BMI, waist/height ratio, waist/hip ratio, systolic and diastolic blood pressure, total cholesterol, triglycerides, LDL-cholesterol, HDL-cholesterol using general linear regression models (backward analysis). Statistical significance was taken as p<0.05. All statistical analysis was performed using the Statistical Package for Social Sciences (SPSS/Windows version 11•0, SPSS Inc., Chicago, IL, USA).

## RESULTS

The characteristics of the study population are shown in Table 1. Both the TS group and control group showed no significant differences in terms of age, weight, systolic and diastolic blood pressure, LDL-cholesterol, fasting insulin, insulin sensitivity indices and cIMT. Subjects in the TS group were significantly shorter than the controls (125.3±14.7 cm versus 135.9±16.2, p=0.021) and had significantly higher BMI (20.0±4.4 versus 16.9±3.0, p=0.007), waist/height (0.5±0.05 versus 0.4±0.02, p=0.001) and waist/hip (0.9±0.06 versus 0.87±0.05, p=0.04) ratios. Total cholesterol, HDL-cholesterol and triglyceride levels were significantly elevated in children with TS, whereas LDL-cholesterol was only slightly higher than the controls without being significant. Fasting glucose was significantly higher in the TS group (92.7±7.5 versus 86.7±8.2 mg/dL, p=0.01). However, differences in fasting insulin and insulin sensitivity indices were non-significant between the groups.

There was no significant difference between the groups for cIMT (0.373±0.06 versus 0.377±0.132 mm; p=0.901). Table 2 shows the correlations between cIMT and other cardiovascular risk factors in children with TS. When cIMT was considered as a continuous variable in the whole population of the TS group, it was found to be significantly positively correlated in univariate analysis with systolic blood pressure (r=0.430, p=0.036), LDL-cholesterol (r=0.435, p=0.034) and FGIR (r=0.376, p=0.07) and significantly negatively correlated with HDL-cholesterol (r=–0.518, p=0.01).

In the control group there was a significant relationship between weight (r=0.413, p=0.045), BMI (r=0.535, p=0.007) and waist/ hip ratio (r=–0.469, p=0.021). There were no significant relationships between cIMT and other clinical and laboratory parameters in this group.

**Figure 1:**
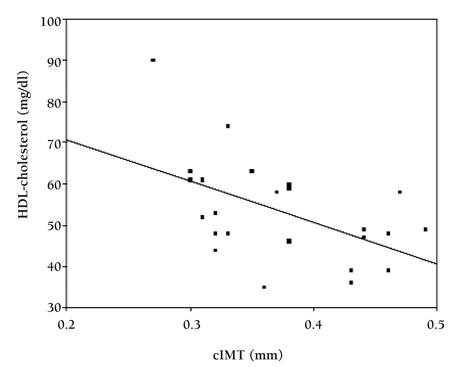
Association between carotid artery intima media thickness (cIMT) and HDL-cholesterol levels in children with Turner syndrome

In the regression analysis, HDL-cholesterol was negatively correlated with increased cIMT (β=-0.518, p=0.01, R^2^=26.8%) even after adjusting for age, weight, BMI, waist/height ratio, waist/hip ratio, systolic and diastolic blood pressure, total cholesterol, triglycerides, LDL-cholesterol, fasting glucose and insulin, insulin sensitivity indices as cofactors with the total variance explained being 26.8%. HOMA-IR, QUICK-I and FGIR were not significantly correlated with cIMT after adjusting for atherosclerotic risk factors. Figure 1 shows the correlation between HDL-cholesterol and cIMT.

**Table 1:**
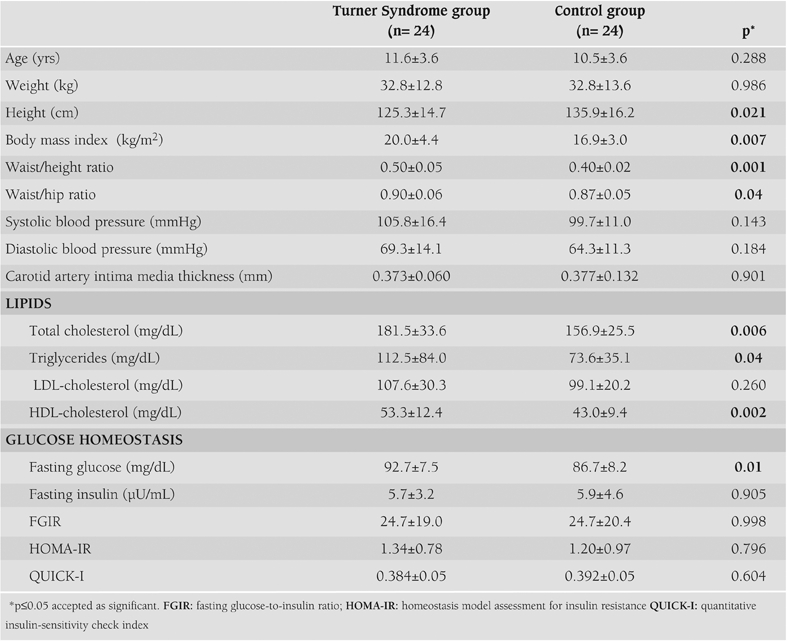
The clinical and metabolic characteristics of the Turner Syndrome and control groups (mean ±SD values)

**Table 2:**
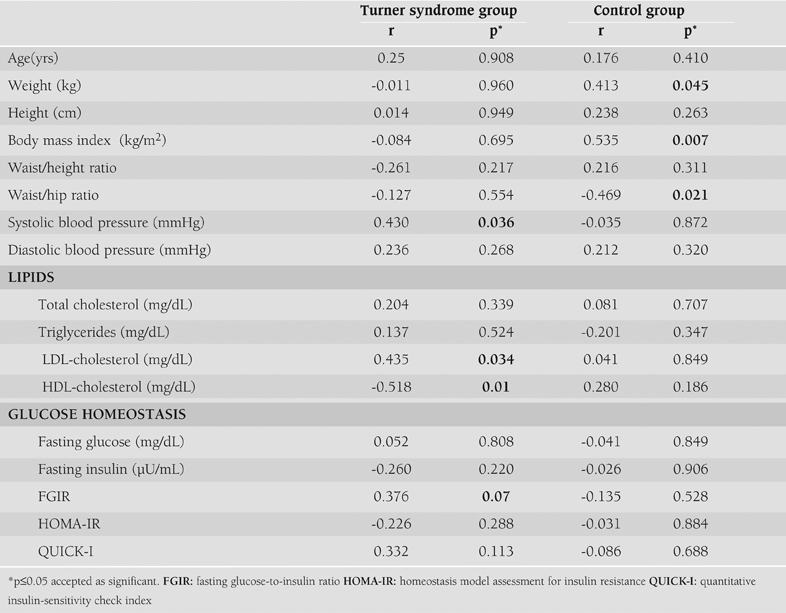
Relationships between carotid artery intima media thickness, insulin sensitivity indices and other cardiovascular risk factors in children with Turner Syndrome and in the control group

## DISCUSSION

High risk factors for atherosclerosis were defined as obesity, diabetes, hypertension and dyslipidaemia in adults with TS. However, up to now, there was a lack of understanding of the risk factors for atherosclerosis in children with TS.

Otsberg JE et al(22) demonstrated the widespread structural vascular differences in adult women with TS characterized by enlargement of conduit arteries and increased carotid intima thickening, compared with normal controls. In the present study, we found no difference between the TS and the control group in terms of cIMT. However, we showed that there were several important relationships between cIMT and cardiovascular risk factors in the TS group.

Coronary heart disease is the most common cause of death in both women and men, and adults with TS seem to have several risk factors for its early development.(23) The risk of hypertension is three-fold in TS patients when compared to the general population.(3) The mechanisms leading to hypertension in these girls are unknown and not clearly related to the cardiac or renal congenital abnormalities.(24) Hypertension has been described with increased frequency in patients with TS even in the absence of aortic coarctation and obvious structural renal abnormalities or history of recurrent urinary infections.(25, 26) In addition, in patients with TS, high levels of triglycerides may be accompanied by high blood pressure.(23) In the present study, the TS group had higher blood pressure measurements than the control group, but the difference was not of statistical significance. However, there was a significant relation between cIMT and systolic blood pressure in the TS group.

In the present study, we found higher levels of lipids in the TS group compared to the control group. Garden et al(27) suggested that hypercholesterolemia occurs in 50% of women with TS over 21 years of age.(27) In a group of women with TS who were tested, hypercholesterolemia was diagnosed in nearly 37% of females, a frequency which was twice as great as in the general population.(28) Analysis of large prospective studies revealed that to assess the risk among women, the HDL-cholesterol level seems to play an essential role. HDL-cholesterol levels below 40 mg/dl (<1.0 mmol/l), which is an indisputable cardiovascular risk factor, were observed in 25% of the population with TS and in 5% of the healthy population. This five- fold difference confirms the suspicion that young women with TS are also more exposed to this cardiovascular risk factor.(29, 30) Ostberg et al(31) used women with premature ovarian failure as controls, in addition to normal controls, and found an essentially comparable cholesterol profile, including triglycerides, to the cholesterol profile in TS, while normal controls had lower levels of cholesterol. Van et al(32), using the same approach with a premature ovarian failure control group, found an elevated cholesterol profile in TS, with a reduced LDL size, suggesting a more atherogenic lipid profile.(32) These factors contribute to the increased patients’ risk of cardiovascular disease.

cIMT is affected by many factors including serum lipids. Autopsy studies in children have also shown a significant relationship between serum cholesterol concentration and early atherosclerotic lesions.(33, 34) Case-control studies of children and young adults demonstrate that familial hypercholesterolemia and borderline hypertension are associated with greater cIMT.(7, 10) In the present study, serum lipid levels (total cholesterol and triglycerides) in TS children were significantly higher than those of healthy subjects. Although HDL-cholesterol level was slightly but significantly higher in TS children than in the controls, we found a significant positive relation between cIMT and LDL-cholesterol and a negative correlation between cIMT and HDL-cholesterol levels, which might have implications for future co-morbidities. Multiple linear regression analysis showed that cIMT had a quantitative relationship with HDL-cholesterol, suggesting that it may be a possible risk factor for atherosclerosis. Although TS girls had higher BMI levels, this correlation was valid after adjusting for BMI.

There are several abnormalities in glucose metabolism in TS and they include an increased frequency of abnormal glucose tolerance, hyperinsulinaemia and reduced insulin sensitivity. Type 2 diabetes mellitus has been diagnosed two to four times more often among women with TS than in the general population, moreover, it is being detected at a younger age.(35, 37) Women with TS have been shown to be insulin resistant which in itself has been implicated in the development of coronary artery disease.(35, 38) A large proportion of adolescent and adult TS patients exhibit impaired glucose tolerance or overt type 2 diabetes during an oral glucose tolerance test.(39, 40) As in the general population, excess weight and an abnormal lipid profile, in particular excess triglyceride levels, worsen insulin sensitivity. In the present study, we showed that the TS group had significantly elevated fasting glucose levels; however, there was no difference between the groups for insulin sensitivity indices. Previous studies have indicated that type 2 diabetes mellitus occurs with increased frequency in TS and this has been shown to affect cardiovascular risk adversely in females.(36, 41) In the present study, we did not detect glucose intolerance in the TS group diagnosed during childhood. The number of subjects is relatively small which might explain these non significant results. Disorders of glucose tolerance, type 2 diabetes, dyslipidaemia, obesity and liver dysfunction often are delayed until adulthood.(29, 42) In the present study, we were unable to show a relationship between fasting glucose-insulin levels and cIMT. These observations suggest that elevated fasting glucose-insulin levels and insulin sensitivity indices may not affect cIMT at younger ages.

Women with TS usually present with increased BMI and waist/hip ratio values.(28) In the present study BMI levels were significantly higher in the TS group than in healthy children. We did not find a significant relationship between anthropometric data and cIMT. The reason for this negative result might be the fact that our subjects had not very high BMI measurements as in obesity.

In conclusion, systolic blood pressure and dyslipidaemia might be associated with early atherosclerosis in children with TS. On the basis of these considerations, in childhood, a fasting lipid profile should be regularly checked in the follow-up of TS patients.
